# Behavioral and Neuropsychological Correlates of Emotion Regulation via Attentional Deployment: An Expanded Replication

**DOI:** 10.5964/ejop.15803

**Published:** 2025-08-29

**Authors:** Christian Salas, Nicolas Núñez, Luz María Pozo, Marko Bremer, Daniel Rojas-Líbano

**Affiliations:** 1 Centro de Estudios en Neurociencia Humana y Neuropsicología (CENHN). Facultad de Psicología, Universidad Diego Portales. Santiago, Chile; 2 Programa de Magíster en Neurociencia Social. Facultad de Psicología, Universidad Diego Portales. Santiago, Chile; Lancaster University, Lancaster, United Kingdom

**Keywords:** emotion regulation, attentional deployment, Attention Network Test, process model of emotion regulation

## Abstract

Attentional deployment (AD) constitutes an emotion regulation (ER) strategy that shifts the attentional focus to modulate the emotional experience. There are very few experimental paradigms that can study AD. One such task studies AD by using emotional images with zones of focus within them, to manipulate visual attention toward arousing or non-arousing portions of the scene. However, this task has only been implemented with participants inside a scanner and has no replications beyond the work of the original research group. In the present study, we replicated and extended a previously introduced AD task, implementing it with a sample of 55 adult participants. Our sample performed the task in a regular laboratory setting, including eye-tracking to monitor instruction following, and in addition, participants completed an attentional test. We replicated the original AD effect in a new population sample, although we found a lower effect size. We conceived and computed an estimate of AD abilities by comparing intensity and valence ratings across attentional conditions. We also analyzed the association between attention measured through the Attention Network Test (ANT) and AD capacities and found no relationship. The task can be used in the laboratory to analyze the AD process. Our replication and expansion of the AD task provide valuable insights into the behavioral and neuropsychological correlates of ER strategies.

The interaction between affect and cognition has been a long-standing area of scientific interest and debate (Forgas, 2008). Most of this debate has focused on how affect confers value to, and modulates, cognitive processes such as perception, memory, or thinking (Bower et al., 1983; Clore & Schiller, 2016; Isen, 1984). Less attention, however, has been given to understanding how cognition influences emotional responses. Emotion regulation (ER) refers to the processes by which individuals influence which emotions they have, when they have them, and how they experience and express these emotions (Gross, 1998). ER has been defined as a top-down process, largely conscious and dependent on cognitive control (McRae & Gross, 2020). Several studies have offered neuropsychological data to suggest that the regulation of emotion via the use of ER strategies requires specific cognitive processes, such as inhibition, working memory, and verbal fluency (Salas et al., 2014, 2016). In consequence, the study of ER and ER strategies can bring some relevant insights into the affect-cognition interaction.

A framework that has structured research on ER is the “process-model”, which proposes five intrinsic strategies often used to regulate emotions (Gross, 1998): situation selection, situation modification, attentional deployment, cognitive reappraisal, and response modulation. Despite the explosive recent growth in ER research, most efforts have focused on cognitive reappraisal (McRae & Gross, 2020) with other strategies receiving less attention. Here, we focus on *Attentional Deployment* (AD), which involves the manipulation of the attentional focus during an emotional situation to downregulate negative emotional experience. Even though AD has been described as emerging early during development (Posner et al., 2014), its behavioral and neuropsychological correlates are still being investigated.

A variety of emotion elicitation procedures with images have been used to study AD in the laboratory (Dunning & Hajcak, 2009; Ferri et al., 2013, 2016). In 2009, Dunning and Hajcak devised a task by superimposing or removing circles around specific areas of negatively valenced pictures to manipulate the participants’ focus of attention. They showed this manipulation modulated the amplitude of electroencephalographic markers of emotional processing (Dunning & Hajcak, 2009). Later, Ferri and colleagues designed an AD task based on similar principles, where participants had to focus their attention within circles placed on emotionally arousing sections or on non-arousing areas of the images. Subsequently, participants were asked to provide emotional ratings, which allowed the assessment of AD by rating comparisons across attentional conditions (Ferri et al., 2013, 2016). The authors showed interactions between the amygdala and frontoparietal regions during AD, assessed by functional magnetic resonance imaging. These data have offered valuable information regarding AD’s neural basis and have shown that this ER strategy can be studied in the laboratory.

Interestingly, to date, we know of no reports or independent studies replicating the AD task presented originally by Ferri et al. (2013). A current challenge in psychology and neurosciences is to replicate and extend its findings to diverse population samples and contexts (Kopal et al., 2023), to evaluate the generalizability of our models and empirical effects. In this regard, we currently lack data from one of the very few experimental tasks that assesses AD in the laboratory, and therefore we do not know to what extent previous results apply to different population samples. In addition, we still know little about AD’s behavioral and neuropsychological correlates. For example, we would like to know the response times people require to report their emotional experiences. How long does it take for people to make these decisions? Is the response time influenced by the valence and arousal of negative pictures? How does the attentional focus affect decision time? We also would like to know the size of the experimental effect of AD: How different is the emotional response to an unpleasant image under different attentional conditions? This is also related to the need to have a readout variable from the task that would serve as an estimate of the AD capacities.

As for the neuropsychological correlates, it has been assumed that AD requires several basic attentional and executive processes. AD would require the ability to sustain attention (Urry & Gross, 2010) and the capacity to decouple attention from emotional stimuli (Compton, 2000). Despite the interest in the literature on the role of cognitive processes in AD, few articles have offered data on this matter, most of them not using AD experimental paradigms. For example, it has been reported that older adults with good executive abilities (measured with the Attention Network Test (ANT), exhibit better resistance to declines in mood (Isaacowitz et al., 2009). An association has also been described between the activation of the orienting attentional network and the subjective report of emotion regulation (Xiu et al., 2018). All this evidence suggests a potential role of attentional abilities in AD. Consequently, exploring the relationship between attentional components, or attentional networks, and AD would be extremely informative for the field of ER specifically, and for the study of cognition-affect interactions more generally.

The present article aims to achieve three primary objectives: Firstly, it seeks to replicate the AD experimental task originally introduced by Ferri et al. (2013), which, to the best of our knowledge, has not been replicated beyond the original research group or implemented with participants outside a scanner. Secondly, the study aims to behaviorally explore AD ability in detail through the analysis of the response time of emotional ratings. Lastly, the study endeavors to investigate the relationship between attentional abilities, as measured by the ANT, and AD performance.

## Method

### Sample Size

We based our calculation of sample size on the publication that we used as a reference to build the task and that we are replicating here (Ferri et al., 2013). They used *n* = 41 participants (Study 1). There, we focused on the size of effect that the attentional manipulation had on participants’ emotional ratings. Ferri et al. reported a rating of *M* = 3.22, *SD* = 0.77 for unpleasant images with arousing focus, and a rating of *M* = 2.11, *SD* = 0.75 for unpleasant images with non-arousing focus. This results in an effect size of *g* = 1.4. Using this effect size, we used the software G*Power 3.1 to estimate sample size considering a matched pairs *t*-test, one tail, α = 0.05, and a power of 0.95. This calculation resulted in a sample size of *n* = 8. In a second report that used the task, sample size was *n* = 51 and there was no report of rating data to calculate effect size (Ferri et al., 2016). Given that it is not a recommended practice to use estimates of effect size from isolated reports, and that published effect size estimates tend to be large and misleading (Button et al., 2013; Gelman & Carlin, 2014), we simply set out to have a sample size larger than the original studies (i.e., *n* = 41 and *n* = 51). Thus, we ended data collection at *n* = 55.

### Participants

A total of 55 participants completed the task (32 female; Age: *M* = 21.9, *SD* = 4.1 years). Participants were recruited through printed posts and social media. The data collection process occurred in two different periods. The first occurred during 2019 (*n* = 39 participants). The second occurred in 2022 (*n* = 16 participants). Our inclusion criteria were to be older than 18 years old and to have completed secondary education. The exclusion criteria were a refusal to sign the written consent or a diagnosis of a neurological condition. The institutional Ethics Committee reviewed and approved the study. All participants signed a written consent form for participation.

### Task and Procedure

All data was collected at the same location, in our laboratory. Participants performed the task individually in a sound-proof, dimly lit experimental room. We used a computer screen View Sonic XG2402, with a spatial resolution of 1920 x 1080 pixels and dimensions of 53.4 cm (width) and 30.1 cm (height) to present the instructions and images throughout the task. After reviewing and signing the informed consent, the entire procedure was explained to the participants. After this explanation, and before commencing the task properly, they were familiarized with the task by executing five exercise trials that used images that were not part of the task.

**Figure 1:**
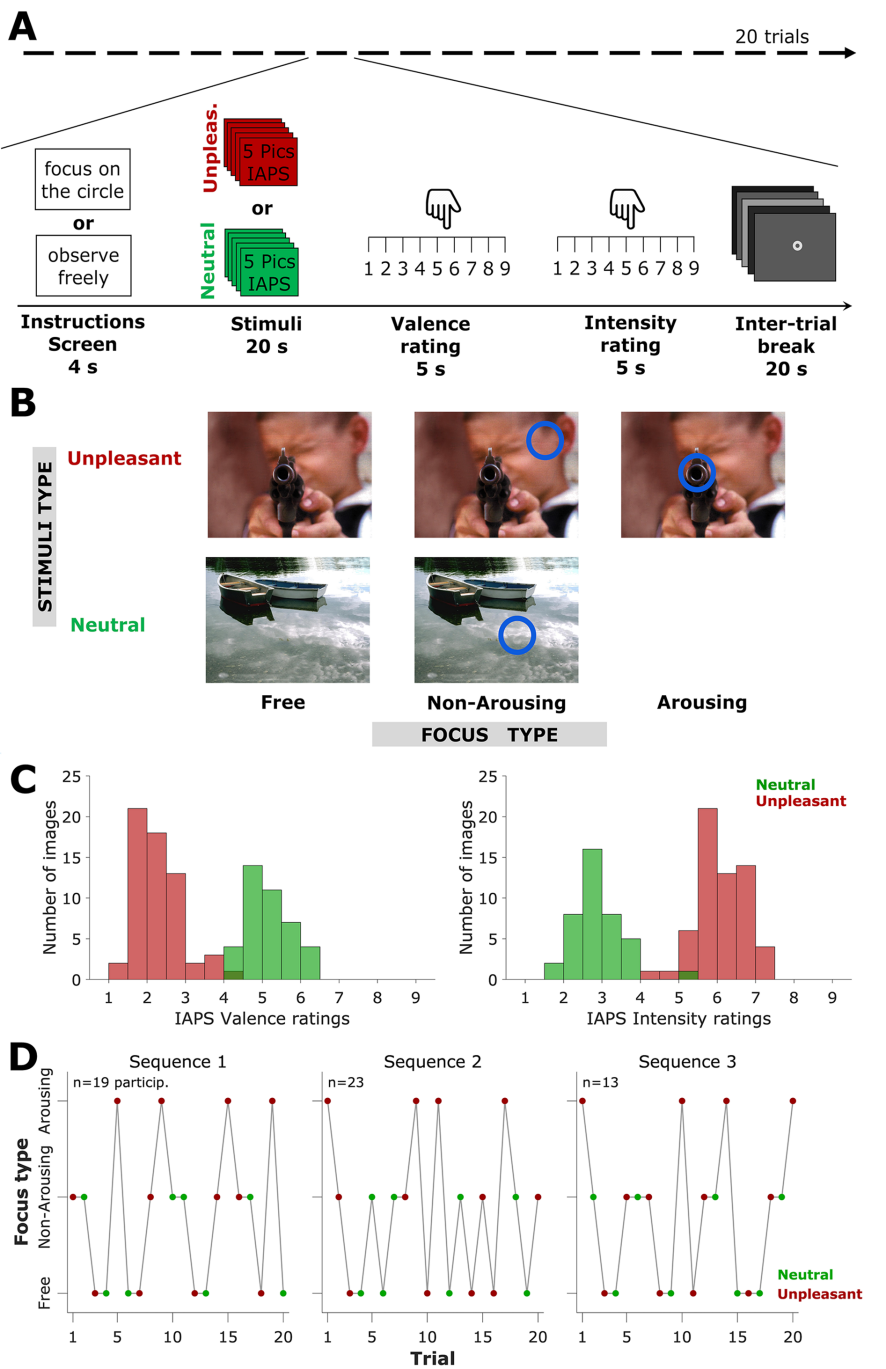
Task Structure, Stimuli and Focus Types, and Trial Sequences *Note*. A. Task and trial structure. Each trial began with an instructions screen, indicating to the participant to focus on the blue circle or to observe it freely. After instructions, stimuli (i.e., five pictures from IAPS) were presented, which could be neutral or negative. Then, the participant had to rate the stimuli in Valence and Intensity. Then, five grey color screens were presented before the next trial. B. Examples of stimuli and focus types. Stimuli could be unpleasant or neutral, and they could have no focus (‘free’), a focus on a non-arousing area of the image, or on an arousing area. Neutral stimuli did not have arousing areas. C. Valences and Intensities of the stimuli used. Left: distributions of valence ratings of the images, according to IAPS data, parsed by type: unpleasant (red) and neutral (green). Right: corresponding data for intensity ratings. D. Trial sequences. Each participant was randomly assigned to one out of three possible sequences, constructed not to contain successive trials of the same type. Numbers on top are the participants assigned to each sequence.

The AD task adopted the procedures described by Ferri and collaborators (Ferri et al., 2013). Our main changes to the task were two: first, participants provided emotional ratings using the original Manikin 1 to 9 scale (instead of the 1 to 4 scale used by Ferri et al.) and second, participants were asked to provide two ratings, one of emotional intensity and one of emotional valence (instead of intensity only as in Ferri et al.). See Figure 1A.

Thus, depending on focus conditions and stimuli (i.e., image) types, each trial belonged to one out of five possible categories (see Figure 1B). Stimuli could be neutral or unpleasant, and they were combined with three focus conditions. Unpleasant stimuli were paired with one of the following conditions: focus-free (no circle on the image), arousing focus (circle on an arousing part of the image), and non-arousing focus (circle on a non-arousing part of the image). Neutral stimuli, by definition, did not have arousing parts, and therefore, they were paired only with focus-free or non-arousing focus. See Figure 1B for an example. The 100 IAPS images used in this task were identical to the ones employed in previous studies (Ferri et al., 2013, 2016; Hajcak et al., 2009). Stimuli were either unpleasant (low valence, high intensity) or neutral (higher valence, lower intensity). Of the 100 stimuli, 60 were unpleasant, with low valence on the 1 to 9 scale (*M* = 2.28, *SD* = 1.47), and 40 were neutral, with higher valence (*M* = 5.14, *SD* = 1.28). Conversely, unpleasant stimuli had a significantly higher level of intensity on the 1 to 9 scale (*M* = 6.05, *SD* = 2.25) compared to neutral stimuli (*M* = 2.91, *SD* = 1.92). See Figure 1C for the distributions of valence and intensity values reported for the set of stimuli.

Each trial began by presenting the instructions written on the screen for 4 seconds. Instructions were “focus your attention on the circle” or “observe the image freely”. After instructions, five images of the same type (neutral or unpleasant) were presented for 4 seconds each. Then, a screen containing an image with Numbers 1 to 9 appeared, with the instruction “Rate the valence, from 1 to 9”. Below the instruction, an image depicting the Manikin figures (Lang et al., 2008) alongside a horizontal scale showing Numbers 1 to 9. Below Number 1 was a text: “Negative”, and below Number 9 was a text: “Positive”. The participant had 5 seconds to complete the rating. They did not have the instruction to answer quickly. Participants delivered their responses through a regular computer keyboard. After delivering the response, the number corresponding to the assigned rating was shown on the screen for 0.35 seconds. If the participant did not complete it, the next screen appeared, containing instructions for Intensity rating. The procedure to record the participant’s intensity rating was similar to the one for valence. After participant ratings, a small fixation circle was presented at the center of the screen. This fixation circle was kept through five homogeneous gray backgrounds: 40%, 10%, 50%, 30%, and 20% of light saturation, which lasted 4 seconds each. After the sequence was completed, a new trial started. The task ended after 20 trials. One out of three possible trial sequences was randomly assigned to each participant to prevent possible confounding order effects (see Figure 1D). See Table 1 for the details of these sequences. Due to the random assignment procedure, we ended up with different numbers of participants in each sequence.

**Table 1:** Sequences of Images Used as Stimuli in the Task

	Sequence 1		Sequence 2		Sequence 3	
Trial	Image	Stimulus-Focus	Image	Stimulus-Focus	Image	Stimulus-Focus
1	9570	Unpl-NonArous	6555	Unpl-Arous	9252	Unpl-Arous
1	8480	Unpl-NonArous	3060	Unpl-Arous	6260	Unpl-Arous
1	3181	Unpl-NonArous	3017	Unpl-Arous	6571	Unpl-Arous
1	9584	Unpl-NonArous	9040	Unpl-Arous	3016	Unpl-Arous
1	3015	Unpl-NonArous	8480	Unpl-Arous	6313	Unpl-Arous
2	7100	Neut-NonArous	6312	Unpl-NonArous	7490	Neut-NonArous
2	5250	Neut-NonArous	9428	Unpl-NonArous	2880	Neut-NonArous
2	7550	Neut-NonArous	6370	Unpl-NonArous	7550	Neut-NonArous
2	7950	Neut-NonArous	3212	Unpl-NonArous	7217	Neut-NonArous
2	7490	Neut-NonArous	3530	Unpl-NonArous	5875	Neut-NonArous
3	9435	Unpl-Free	9435	Unpl-Free	9265	Unpl-Free
3	9252	Unpl-Free	9403	Unpl-Free	9300	Unpl-Free
3	9430	Unpl-Free	6560	Unpl-Free	9430	Unpl-Free
3	6370	Unpl-Free	9410	Unpl-Free	3053	Unpl-Free
3	9403	Unpl-Free	6260	Unpl-Free	6560	Unpl-Free
4	2880	Neut-Free	7004	Neut-Free	2393	Neut-Free
4	7233	Neut-Free	5875	Neut-Free	2320	Neut-Free
4	2383	Neut-Free	2270	Neut-Free	7700	Neut-Free
4	2440	Neut-Free	2102	Neut-Free	2440	Neut-Free
4	7285	Neut-Free	5250	Neut-Free	5250	Neut-Free
5	3530	Unpl-Arous	7140	Neut-NonArous	3261	Unpl-NonArous
5	2717	Unpl-Arous	7705	Neut-NonArous	3530	Unpl-NonArous
5	2800	Unpl-Arous	7550	Neut-NonArous	8480	Unpl-NonArous
5	6242	Unpl-Arous	2745.1	Neut-NonArous	3005.1	Unpl-NonArous
5	3060	Unpl-Arous	2320	Neut-NonArous	3010	Unpl-NonArous
6	7090	Neut-Free	2580	Neut-Free	7175	Neut-NonArous
6	7025	Neut-Free	7491	Neut-Free	7595	Neut-NonArous
6	7560	Neut-Free	2440	Neut-Free	2102	Neut-NonArous
6	5530	Neut-Free	2880	Neut-Free	2235	Neut-NonArous
6	2270	Neut-Free	7700	Neut-Free	7150	Neut-NonArous
7	3030	Unpl-Free	5740	Neut-NonArous	3212	Unpl-NonArous
7	3261	Unpl-Free	7090	Neut-NonArous	9635.1	Unpl-NonArous
7	3266	Unpl-Free	7100	Neut-NonArous	3063	Unpl-NonArous
7	9410	Unpl-Free	7490	Neut-NonArous	9428	Unpl-NonArous
7	6571	Unpl-Free	2206	Neut-NonArous	3015	Unpl-NonArous
8	3212	Unpl-NonArous	6570.1	Unpl-NonArous	3030	Unpl-Free
8	3016	Unpl-NonArous	3181	Unpl-NonArous	6555	Unpl-Free
8	9810	Unpl-NonArous	9571	Unpl-NonArous	9571	Unpl-Free
8	3005.1	Unpl-NonArous	6550	Unpl-NonArous	3220	Unpl-Free
8	9433	Unpl-NonArous	9570	Unpl-NonArous	9410	Unpl-Free
9	2811	Unpl-Arous	9405	Unpl-Arous	7285	Neut-Free
9	3225	Unpl-Arous	2717	Unpl-Arous	7950	Neut-Free
9	6260	Unpl-Arous	3213	Unpl-Arous	2190	Neut-Free
9	3010	Unpl-Arous	9430	Unpl-Arous	2745.1	Neut-Free
9	2703	Unpl-Arous	3220	Unpl-Arous	7560	Neut-Free
10	7175	Neut-NonArous	3015	Unpl-Free	9405	Unpl-Arous
10	5390	Neut-NonArous	2811	Unpl-Free	2811	Unpl-Arous
10	7217	Neut-NonArous	9252	Unpl-Free	3213	Unpl-Arous
10	2580	Neut-NonArous	9400	Unpl-Free	9584	Unpl-Arous
10	7491	Neut-NonArous	3016	Unpl-Free	6370	Unpl-Arous
11	2235	Neut-NonArous	9584	Unpl-Arous	2800	Unpl-Free
11	2745.1	Neut-NonArous	9300	Unpl-Arous	6242	Unpl-Free
11	7150	Neut-NonArous	3550	Unpl-Arous	9435	Unpl-Free
11	2190	Neut-NonArous	9433	Unpl-Arous	3181	Unpl-Free
11	7020	Neut-NonArous	6022	Unpl-Arous	9253	Unpl-Free
12	9405	Unpl-Free	7000	Neut-Free	6415	Unpl-NonArous
12	6570.1	Unpl-Free	7020	Neut-Free	6022	Unpl-NonArous
12	6555	Unpl-Free	2383	Neut-Free	3195	Unpl-NonArous
12	3220	Unpl-Free	5530	Neut-Free	3225	Unpl-NonArous
12	3017	Unpl-Free	7002	Neut-Free	2730	Unpl-NonArous
13	7010	Neut-Free	7175	Neut-NonArous	7491	Neut-NonArous
13	7140	Neut-Free	7560	Neut-NonArous	7000	Neut-NonArous
13	2980	Neut-Free	7950	Neut-NonArous	2383	Neut-NonArous
13	2320	Neut-Free	2393	Neut-NonArous	5740	Neut-NonArous
13	7000	Neut-Free	7150	Neut-NonArous	7020	Neut-NonArous
14	6831	Unpl-NonArous	6415	Unpl-Free	9403	Unpl-Arous
14	3195	Unpl-NonArous	2800	Unpl-Free	6831	Unpl-Arous
14	3550	Unpl-NonArous	6315	Unpl-Free	9810	Unpl-Arous
14	9040	Unpl-NonArous	3266	Unpl-Free	6312	Unpl-Arous
14	6560	Unpl-NonArous	9253	Unpl-Free	9040	Unpl-Arous
15	9400	Unpl-Arous	3261	Unpl-NonArous	5390	Neut-Free
15	2730	Unpl-Arous	3211	Unpl-NonArous	7705	Neut-Free
15	9300	Unpl-Arous	3063	Unpl-NonArous	7025	Neut-Free
15	9420	Unpl-Arous	6190	Unpl-NonArous	7140	Neut-Free
15	9428	Unpl-Arous	3010	Unpl-NonArous	2206	Neut-Free
16	6550	Unpl-NonArous	9635.1	Unpl-Free	9400	Unpl-Free
16	3213	Unpl-NonArous	2730	Unpl-Free	6550	Unpl-Free
16	9635.1	Unpl-NonArous	9810	Unpl-Free	9433	Unpl-Free
16	6022	Unpl-NonArous	6831	Unpl-Free	6315	Unpl-Free
16	9571	Unpl-NonArous	6242	Unpl-Free	2703	Unpl-Free
17	7004	Neut-NonArous	3005.1	Unpl-Arous	7100	Neut-Free
17	5875	Neut-NonArous	3053	Unpl-Arous	7002	Neut-Free
17	2102	Neut-NonArous	3030	Unpl-Arous	7233	Neut-Free
17	7705	Neut-NonArous	2703	Unpl-Arous	2580	Neut-Free
17	7595	Neut-NonArous	6313	Unpl-Arous	7090	Neut-Free
18	3211	Unpl-Free	7233	Neut-NonArous	2717	Unpl-NonArous
18	6190	Unpl-Free	7010	Neut-NonArous	3266	Unpl-NonArous
18	9265	Unpl-Free	7025	Neut-NonArous	6190	Unpl-NonArous
18	6415	Unpl-Free	7285	Neut-NonArous	9420	Unpl-NonArous
18	9253	Unpl-Free	2980	Neut-NonArous	3550	Unpl-NonArous
19	6313	Unpl-Arous	7595	Neut-Free	5530	Neut-NonArous
19	3053	Unpl-Arous	5390	Neut-Free	2980	Neut-NonArous
19	6315	Unpl-Arous	7217	Neut-Free	7010	Neut-NonArous
19	3063	Unpl-Arous	2235	Neut-Free	2270	Neut-NonArous
19	6312	Unpl-Arous	2190	Neut-Free	7004	Neut-NonArous
20	5740	Neut-Free	9420	Unpl-NonArous	3060	Unpl-Arous
20	7700	Neut-Free	6571	Unpl-NonArous	3211	Unpl-Arous
20	7002	Neut-Free	3195	Unpl-NonArous	3017	Unpl-Arous
20	2393	Neut-Free	3225	Unpl-NonArous	6570.1	Unpl-Arous
20	2206	Neut-Free	9265	Unpl-NonArous	9570	Unpl-Arous

*Note.* Table 1. Possible Trial Sequences. Each participant was randomly assigned to one out of three possible trial sequences, constructed not to contain successive trials of the same type. The combination of the image and focus types determined the trial type. Sequence 1 was assigned to 19 participants, Sequence 2 to 23 participants, and Sequence 3 to 13 participants. For each sequence, the table shows the trial number (each trial consists of the presentation of 5 images), the IAPS image name, and the corresponding Stimulus and Focus type. Neut: Neutral image; Unpl: Unpleasant image; Arous: Arousing focus; NonArous: Non-Arousing focus; Free: focus-free (no focus).

All hardware controls and data acquisition (behavioral and physiological) routines were written in Matlab (Version: 9.6.0.1472908 (R2019a) Update 9), using the Psychophysics Toolbox extension (Brainard, 1997; Kleiner et al., 2007).

### Eye Tracking Recording and Analysis

Eye movement data, used to monitor instruction-following behavior, was acquired using an Eyelink 1000 (SR Research Ltd., Mississauga, Ontario, Canada) with a 500 Hz sampling frequency. Throughout the task, participants sat in front of the computer screen and the eye tracker device and kept their heads in a forehead/chin rest (SR Research Ltd.) placed 56 cm from the screen. Gaze data files were converted to the ASCII format using Eyelink’s EDFConverter, and then analyzed using custom routines written in Matlab (Version: 9.6.0.1472908 (R2019a) Update 9). We discarded gaze data from trials in which gaze detection was lost due to excessive blinks or other software/recording errors (for example). This resulted in completely discarding 12 participants from the statistical descriptions and comparisons.

Gaze behavior was quantified as dwelling time: the percentage of total time spent by the gaze within the focus circle during the image presentation (4 seconds). The raw data contained the (X, Y) screen coordinates where the gaze was located on each sampled data point. We classified each sample by marking whether it was inside or outside the focus circle. This allowed us to count the number of samples inside the circle, divide it by the total number (4 s x 500 samples/s = 2000 samples), and express it as a percentage to obtain the dwelling time. We calculated this dwelling time for each image and the mean of the five images of a trial. This gave us a single value of dwelling time per trial. The overall dwelling time was obtained by averaging dwell times across trials.

### ANT

To estimate the participants’ attentional abilities, we used a computerized version of the ANT, which measures the capacity for alertness, orientation, and attentional executive control (Fan et al., 2002). The efficiency of the alerting network was examined through differences in reaction times in response to a warning signal before the presentation of stimuli. The efficiency of the orienting network was examined through differences in reaction times in response to a cue indicating where a stimulus will appear. The efficiency of the executive control network was examined through differences in reaction times in response to the presentation of a central arrow surrounded by congruent or incongruent indicators (arrows in the same or different directions to the central arrow). The differences in the reaction times associated with using each attentional system were used as a score to evaluate the performance of each attentional network (Alertness, Orientation, and Executive Control Score). For all these measurements, we followed the standard previously published procedures (Fan et al., 2002).

Given that participants completed the ANT after the experimental task, we had two periods of data collection (See Method section, Participants subsection). However, due to technical problems in retrieving the ANT data from the software, we were able to use only the data from the first period. And in this period (*n* = 39), data was lost for 4 participants. Therefore, we have ANT data only for 35 participants.

### Data Analysis

In general, data are summarized by means (*M*), medians (Mdn), standard deviations (*SD*), and inter-quantile range (IQR). For two-group comparisons, we implemented a non-parametrical permutation test based on the *t*-statistic. This allowed us to deal with heterogeneity in our data, which were not always normally distributed. We based our tests on previously published protocols (Ernst, 2004; Maris & Oostenveld, 2007). First, we computed the regular two-sample *t*-test and saved the corresponding *t*-value (i.e., the *t*-statistic value). We then randomly permuted (reorganized) the values from both groups, forming two new groups, and implemented the *t*-test, calculating the new *t*-value and saving it. We repeated this permutation procedure 1500 times and constructed a distribution of the permutation-based *t*-values. We then computed the *p*-value as the proportion of *t*-values in the distribution equal to or larger than the non-permuted t-value. Therefore, we report the non-permuted *t*-value, the corresponding degrees of freedom, and the *p*-value. For each two-group comparison, we also present the Hedges’ *g* statistic as a measure of effect size (Hedges, 1981), which calculates effect size as a difference in means standardized by the pooled standard deviation. Hedges’ *g* value and associated confidence intervals were calculated using a previously available Matlab toolbox (Hentschke & Stüttgen, 2011).

### Transparency and Openness

The Matlab code used for controlling hardware and implementing the task, and all the code used to analyze the data, produce plots, and compute statistics are available at Salas et al. (2025a) and Salas et al. (2025b). All the data collected and used to compute the results presented in this article are available at Rojas Libano (2025). All the image files used as stimuli for the task trials are available at Salas et al. (2025a). We report in the methods section how we determined our sample size, all data exclusions, manipulations, and all study measures. We also report effect sizes and confidence intervals. This study was not preregistered.

## Results

**Figure 2:**
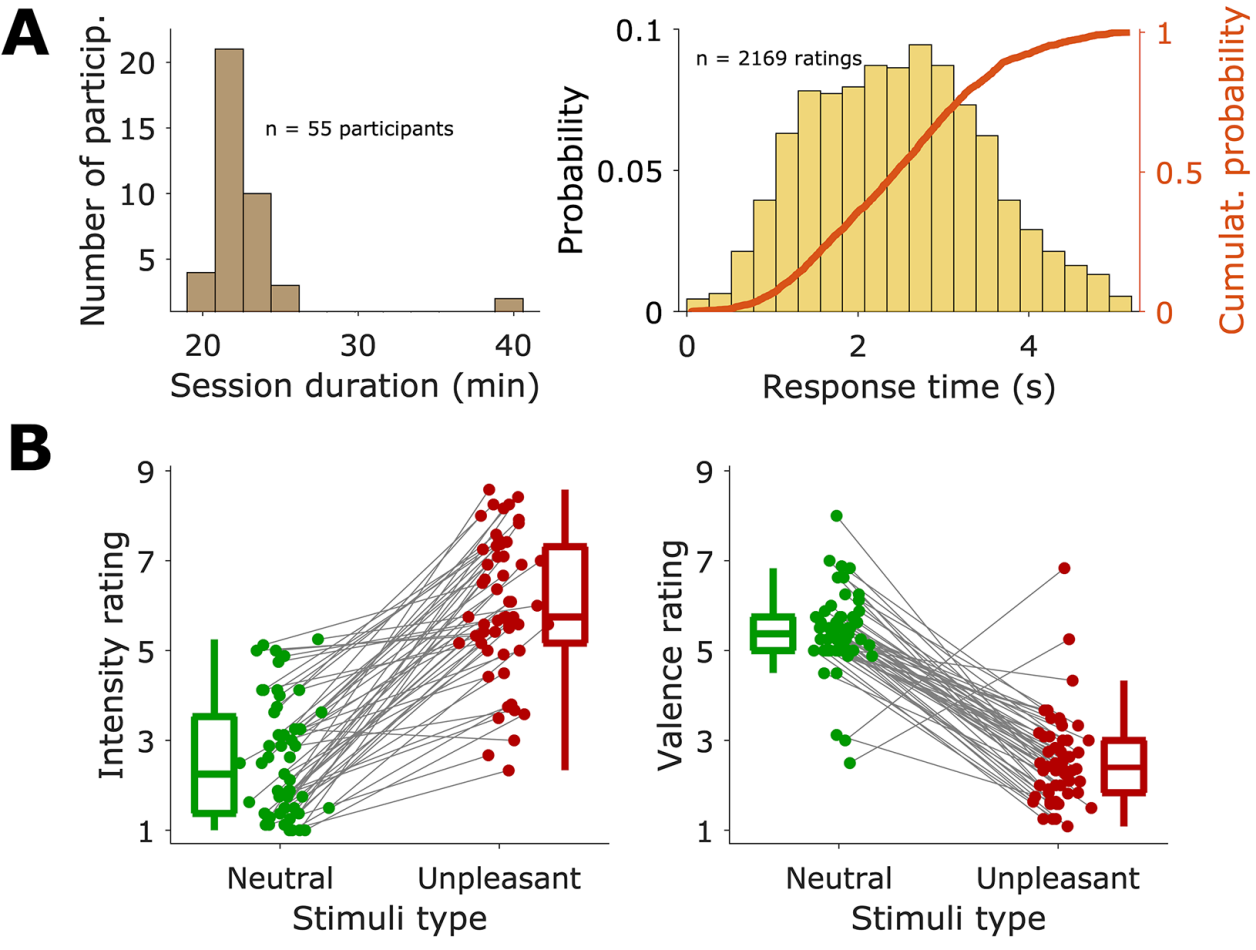
Task Duration, Response Times, and Ratings for Each Image Type *Note.* A. Left: Distribution of the duration of the experimental session for the 55 participants. Right: Distribution of response times, in seconds, for all ratings. 55 participants x 20 trial/participant x 2 ratings/trial = 2200 ratings. The orange curve read in the right y-axis corresponds to the empirical cumulative distribution of these response time values. B. Mean Intensity (left) and Valence (right) ratings for each participant, sorted by image types. Each marker corresponds to the mean of all trials of a given stimulus type for the entire task. Grey lines link markers for the same participant.

Participants completed the task in around 20 minutes (*M* = 23.7, *SD* = 4.6, *n* = 55) (See Figure 2A). In general, participants did not omit response ratings (from a total of 2200 possible ratings (20 trials x 2 ratings/trial = 40 ratings per participant), we collected 2169 (98.6%), with 16 participants omitting 1 rating, 2 participants omitting 2 ratings, 2 participants omitting 3 ratings, and 1 participant omitting 5 ratings). The response time (time between the presentation of the rating screen and the completion of the rating response) for individual ratings of valence and intensity was around 2 seconds (*Mdn* = 2.44 s, *M* = 2.46 s, *SD* = 1.0 s, *n* = 2169 ratings), even when participants had 5 seconds to deliver the response (see Figure 2A). Therefore, these responses, associated with the AD process, were relatively quick.

The responses evoked by the unpleasant images were markedly different from neutral ones, showing that the images elicited emotions in participants (Figure 2B). On the 1 to 9 scale used, emotional intensity ratings were significantly higher for unpleasant than for neutral images (unpleasant: *Mdn* = 5.75, *M* = 5.99, *SD* = 1.56; neutral: *Mdn* = 2.25, *M* = 2.55, *SD* = 1.3), with a large effect size, *t*(54) = 13.57, *p* < .01, *g* = 2.38, 95% *CI* [1.96, 2.96]. Correspondingly, emotional valence ratings on the 1 to 9 scale were lower for unpleasant than neutral images (unpleasant: *Mdn* = 2.42, *M* = 2.5, *SD* = 1; neutral: *Mdn* = 5.38, *M* = 5.4, *SD* = 0.9), with a large effect size, *t*(54) = -13.52, *p* < .001, *g* = -2.97 [-4.52, -2.04]. These results show that the task worked as expected, eliciting in participants emotional responses that were detectable through their subjective reports. These effects were the same for all three trial sequences, showing that they depended on the image types and not the specific sequence of images.

### Gaze Behavior

**Figure 3:**
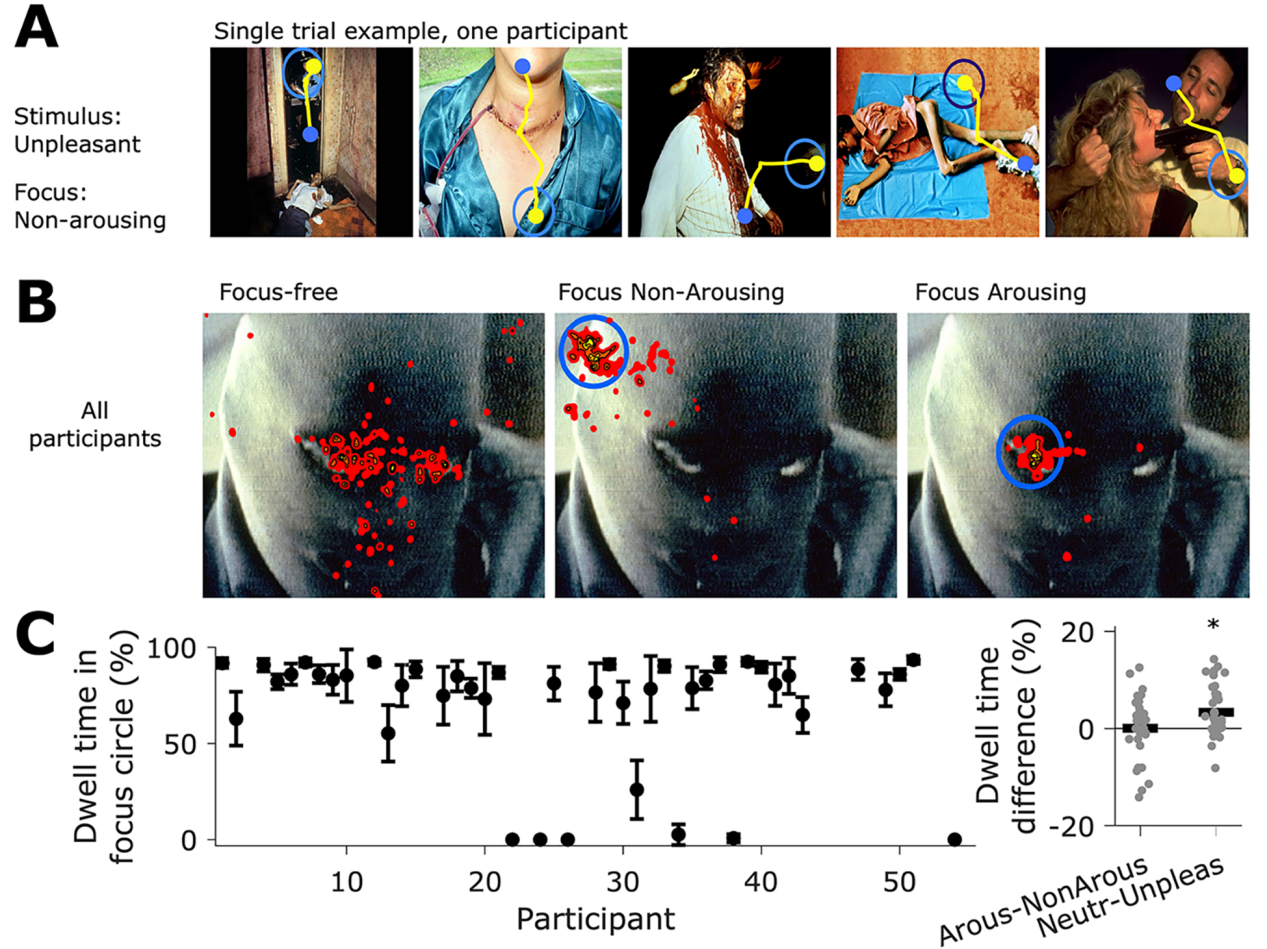
Gaze Data During the Task *Note*. A. Example of gaze trajectory during a single trial. In this trial. images were of a negative type, and the focus was on a non-arousing area of the image. The blue and yellow dots mark the gaze position at the start and end of the image's 4-second period. The yellow line corresponds to the gaze trajectory. Since the images were presented in the same sequence shown, the gaze end position of an image corresponds to the start position in the next image. B. Heat maps of pixels visited by participants’ gaze. Data corresponds to all trials where the image was presented, parsed by the three attentional conditions: focus-free (left), focus on a non-arousing area of the image (center), and focus on an arousing part (right). Spatial data from all participants was summed to obtain each heat map. C. The left plot shows the dwell gaze times in the focus circle as a percentage of the time the image was shown. Dots mark the mean, and error bars represent the standard deviation, where each marker corresponds to a participant. Some participants were excluded because of missing data. The right plot shows the dwell time difference between image types (Neutral minus Unpleasant) and between attentional conditions (Arousing minus Non-Arousing). Each dot represents one participant. The black bars correspond to the mean. * indicates a significant difference compared to a distribution of mean = 0.

We monitored participants’ gaze through eye tracking to observe their instruction-following behavior while viewing the task images. Participants generally followed the instructions given (for some examples see Figure 3A and 3B). The gaze behavior was quantified as the dwelling time of the gaze within the blue circle presented in the images and expressed as a percentage of the total time (4 s) of image presentation. The overall dwelling time was high (*Mdn* = 81.97%, *M* = 69.76%, *SD* = 30.6%, *n* = 43), meaning participants tended to keep their gaze within the circle when required (Figure 3C, left-side plot). When we computed the difference in dwelling time between image types (neutral vs. unpleasant), we found that participants spent slightly less time within the circle for unpleasant than neutral images (Neutral: *Mdn* = 86.3%, *M* = 71.94%, *SD* = 30.95%; Unpleasant: Mdn = 81.87%, *M* = 70.31%, *SD* = 28.96%), with *t*(41)= 4.39, *p* < .01 (see Figure 3C, right-side plot). When we considered different experimental conditions (arousing focus vs. non-arousing focus), differences in dwelling time were smaller (Arousing: *Mdn* = 85.52%, *M* = 76.97%, *SD* = 23.43%; Non-arousing: *Mdn* = 82.43%, *M* = 76.92%, *SD* = 21.19%). In summary, these results show that participants engaged in the task and followed the instructions related to the visual attentional focus, irrespective of image or focus type.

### Attentional Deployment

**Figure 4:**
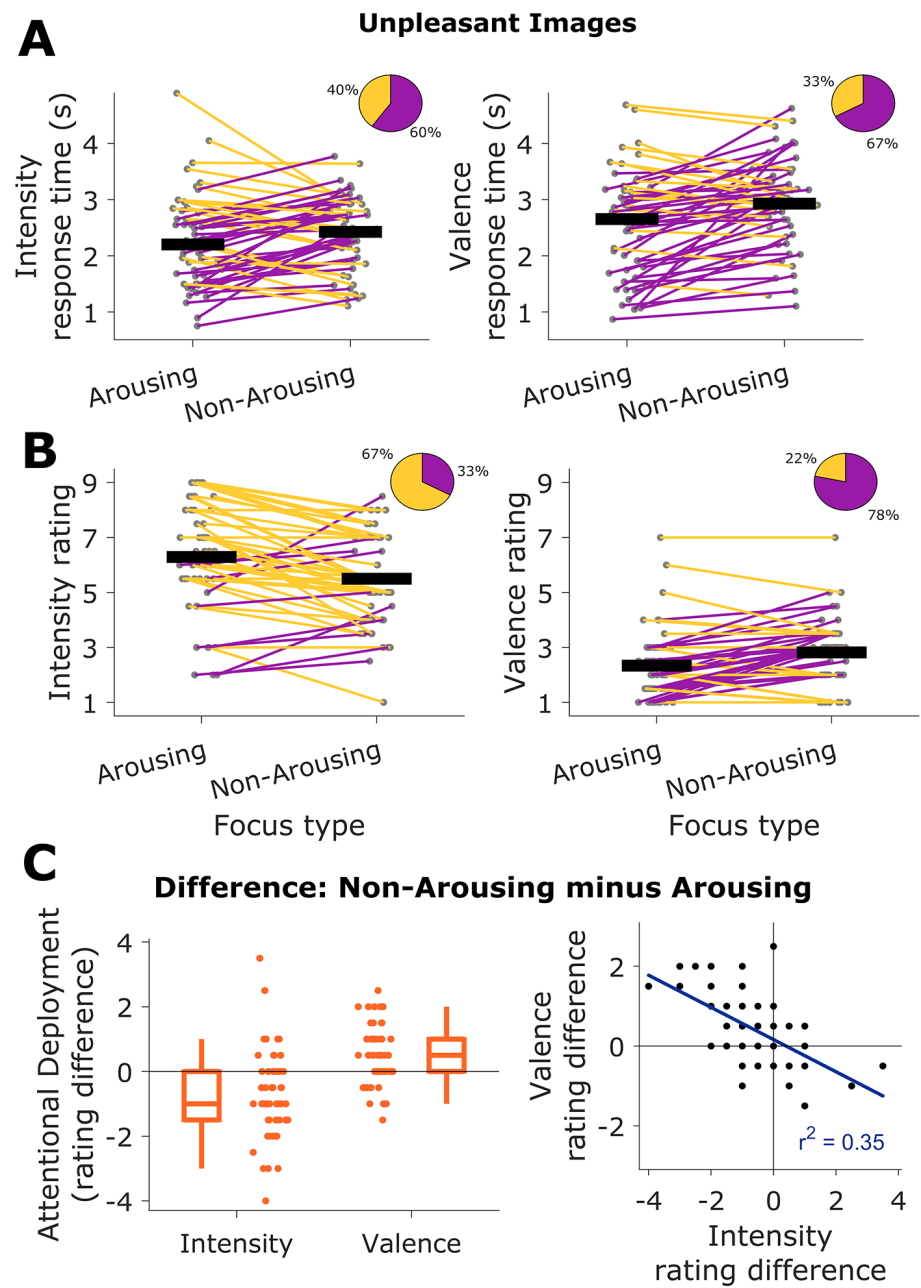
Intensity and Valence Ratings and AD Estimates *Note.* A. Response times for intensity (left) and valence (right) ratings for arousing and non-arousing focus. Black horizontal bars are the group means, and gray circles represent the means of individual participants across the task. Lines represent increases (purple) and decreases (orange) in response times. Pie charts represent the corresponding percentage of participants . B. Intensity (left) and valence (right) ratings for unpleasant images, sorted by focus type. Colors and insets as in A. C. AD estimates. Left: The mean rating for a given focus type was subtracted from the mean rating of the other focus type, separately for intensity and valence. Each circle represents one participant. Boxplots represent the distributions. Right: Same data from the left plot, with rating differences in valence and intensity plotted against each other.

Participants had 5 seconds to provide their responses of emotional ratings. Within this temporal frame, they were faster to produce emotional ratings in the arousing than in the non-arousing focus conditions. This difference was presented for intensity (non-arousing *Mdn* = 2.43 s; arousing *Mdn* = 2.2 s), *t*(54) = -1.8, *p* = .03, *g* = -0.22 [-0.6, 0.16], with 60% of the participants showing faster responses for the arousing focus. The same was observed for valence ratings (non-arousing: *Mdn* = 2.93 s; arousing: *Mdn* = 2.66 s), *t*(54) = -3.8, *p* < .01, *g* = -0.44, [-0.84, -0.09], where 67% of the participants displayed faster responses for the arousing focus (see Figure 4A).

To further characterize the behavioral responses, we then assessed for associations, computing Spearman’s rank correlation coefficient between the ratings’ response times and the value of the ratings, parsed by attentional focus condition. For the relation between intensity response times and intensity ratings, we obtained *rho* = -0.24, *p* = .07 (Non-Arousing focus), and *rho* = -0.51, *p* < .01 (Arousing focus). For the relation between valence response times and valence ratings, we obtained *rho* = 0.19, *p* = .16 (Non-Arousing focus), and *rho* = 0.32, *p* = .02 (Arousing focus). Thus, specifically for the arousing focus condition, we report a small albeit detectable relationship. Under this condition, the larger the intensity rating, the shorter the response time, and conversely, the larger the valence, the larger the response time.

Emotional ratings were expressed on a 1 to 9 scale. For emotional intensity, we found that the non-arousing focus condition elicited lower ratings than the arousing focus, evidencing a regulation of the emotional experience (non-arousing: *M* = 5.5, *SD* = 1.67; arousing: *M* = 6.5; *SD* = 1.99), with *t*(54) = 4.37, *p* < .01, *g* = 0.43 [0.06, 0.86]. Conversely, we found that for emotional valence, the non-arousing focus elicited higher ratings than the arousing focus, again evidencing regulation (non-arousing: *M* = 2.82, *SD* = 1.16; arousing: *M* = 2.34, *SD* = 1.23), with *t*(54) = -3.89, *p* < .01, *g* = -0.4 [-0.85, -0.04]. These results are shown in Figure 4B.

We then computed our AD estimates, constituted by the differences in rating between the non-arousing and arousing focus conditions. The rating difference was calculated as the participant’s mean rating for the non-arousing focus minus the mean rating for the arousing focus. Given that we had ratings for emotional intensity and valence, we computed two estimates, one for each emotional dimension. For emotional intensity, we found a distribution that was centered on negative values, *Mdn* (*IQR*) = -1(1.5), reflecting a decrease in emotional intensity in the presence of a non-arousing focus. The opposite was true for emotional valence, with a distribution of differences centered in positive values, *Mdn* (*IQR*) = 0.5(1), reflecting an increase in valence for the non-arousing focus.

Finally, we estimated the relationship between these two estimates to assess their congruence using the Pearson product-moment correlation coefficient. We found a correlation between intensity and valence rating differences, *r*^2^ = 0.35; *p* < .01. This result implied that, on average, the effects of changing attentional focus (i.e., attentional deployment) were consistent across the two emotional dimensions: the larger the effect in intensity, the larger in valence. Therefore, our task produced valence-based and intensity-based estimates of AD.

We also studied the ratings and rating differences for the images in the focus-free condition. In these cases, participants did not have an instructed attentional focus on the image and were free to explore it visually. Regarding response times, we found no differences between non-arousing focus and focus-free conditions, both for intensity (focus-free *Mdn* = 2.4 s; arousing *Mdn* = 2.2 s), with *t*(54) = 1.42, *p* = .93, *g* = 0.13 [-0.24, 0.53], and valence (focus-free *Mdn* = 2.68 s; arousing *Mdn* = 2.66 s), with *t*(54) = 0.44, *p* = .67, *g* = 0.04 [-0.34, 0.42]. When examining ratings, we found very small differences between focus-free and arousing focus, for intensity (focus-free: *M* = 6.5, *SD* = 1.76; arousing: *M* = 6.5, *SD* = 1.99), *t*(54) = 1.69, *p* = .05, *g* = 0.12 [-0.26, 0.46], and also for valence (focus-free: *M* = 2.1, *SD* = 1.08; arousing: *M* = 2.34, *SD* = 1.23), *t*(54) = -2.72, *p* = .003, *g* = -0.2 [-0.57, 0.15]. These slim differences meant the focus-free attentional condition was similar to the arousing focus one regarding the emotional response it elicited.

### ANT

**Figure 5:**
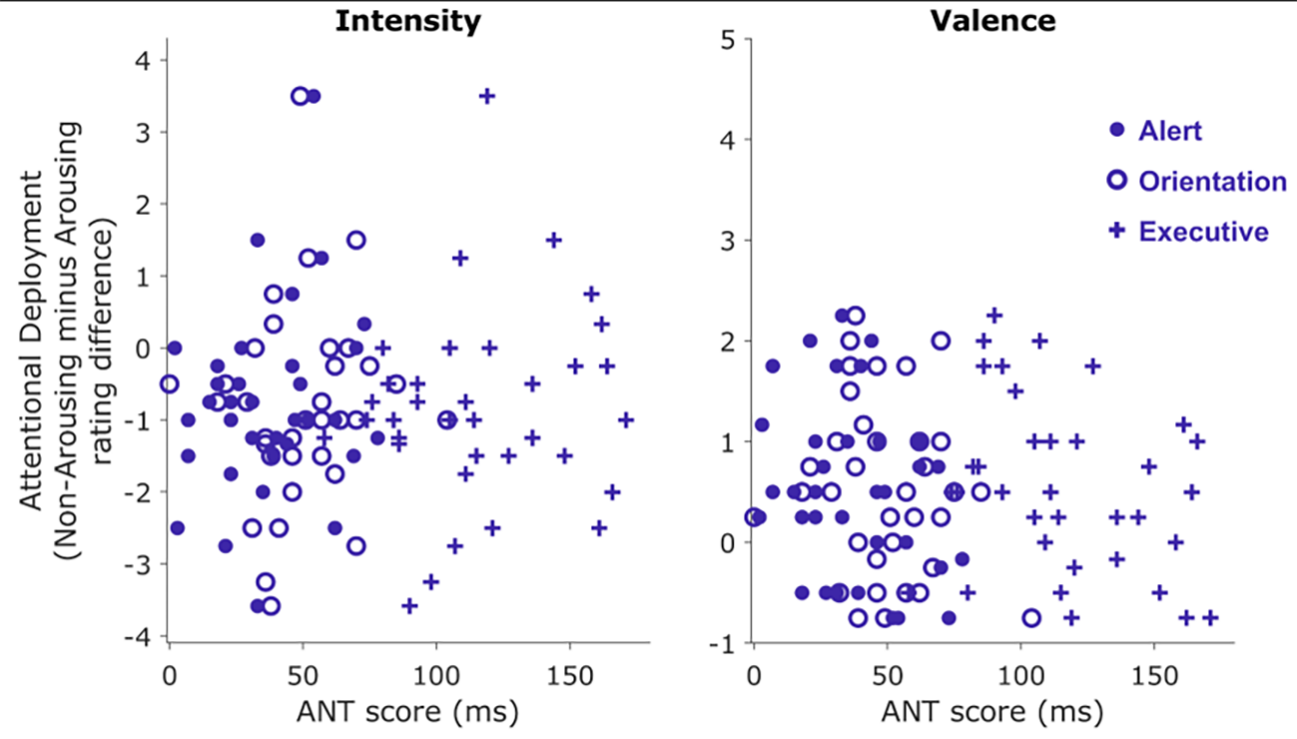
Relationship Between ANT Scores and AD Estimates *Note.* Left: Intensity rating difference as a function of ANT scores, parsed by the attentional networks assessed by the ANT. Right: Valence rating difference as a function of ANT scores, parsed by the attentional networks.

The ANT allowed us to measure performance in three types of attention or attentional systems (Fan et al., 2002). Data from this sample showed a linear increase in the amount of time used by participants, reflecting an increase in the cognitive load of each attentional sub-task; from alerting to orientation to executive: Alerting Network (*Mdn* = 33 ms, *M* = 35.46 ms, *SD* = 22.88 ms), Orientation Network (*Mdn* = 46, *M* = 49.43 ms, *SD* = 20.1 ms), Executive Network (*Mdn* = 111 ms, *M* = 116.16 ms, *SD* = 30.82 ms). When error rates were analyzed, a sharp increase in the percentage of errors in the Executive Network task compared to the Alerting Network and Orienting Network was found: Alerting Network (*Mdn* = 0%, *M* = 0.66%, *SD* = 1.21%), Orientation Network (Mdn = 0%, *M* = 0.26%, *SD* = 0.78%), Executive Network (*Mdn* = 4%, *M* = 4.86%, *SD* = 5.27%). A key question of this study was the relationship between attentional ability -measured by performance on the ANT- and AD ability (AD estimate). Contrary to our hypotheses, no relationship was found between AD ability and performance on attentional tasks (See Figure 5B). We examined the Pearson product-moment correlation coefficient between the intensity-based AD estimate and the Alert Network, *r* = 0.31, *p* = .06, Orientation Network, *r* = 0.11, *p* = .53, and Executive Network, *r* = 0.14, *p* = .43, subdomains of the test. The same was true for the valence-based AD estimate and the Alert Network, *r* = -0.33, *p* = 0.05, Orientation Network, *r* = -0.16, *p* = .34, and Executive Network, *r* = -0.27, *p* = .11, although in this case all correlation values were negative. These results suggest that attentional capacities, as assessed by ANT, do not seem to explain the observed AD ability in our sample of participants.

In summary, we replicated a previously published AD task (Ferri et al., 2013, 2016), extending the sample to a different country, and studied in more detail some of its behavioral and neuropsychological correlates. This is a simple and brief experimental task that requires no more than 20 minutes to complete. In terms of emotional elicitation, our data showed that the task is effective, as emotional intensity ratings increase for unpleasant images compared to neutral ones, and emotional valence ratings are lower for unpleasant images (see Figure 2). In terms of behavioral correlates of the task, we observed for unpleasant images that rating response times were shorter (quicker) for ratings involving images with an arousing, as compared to non-arousing, attentional focus. We also found that, specifically for the arousing focus conditions, intensity ratings were inversely related to response times, and conversely, valence ratings were positively associated with response times. In addition, data from eye tracking showed that participants generally followed instructions, with no significant difference in gaze dwelling time between image types or focus conditions. Regarding estimates of AD, we operationalized it as a rating difference between attentional conditions (Non-Arousing and Arousing focus). We observed that the manipulation of attentional focus generated a decrease in emotional intensity and an increase in emotional valence, consistent with previous reports. As for the neuropsychological correlates of AD, no significant relationships were found between AD ability and performance on any of the ANT subtasks.

## Discussion

This study aimed to replicate a previously published experimental task devised to estimate AD capacities, analyze emotion regulation (AD) behavioral performance in detail, and study the relation between AD and attentional capacities measured through a neuropsychological test. Our results provide several insights into the AD process specifically and the emotion-cognition interaction more generally.

Our task extends the original sample of participants, consistent with English speakers from the USA. In contrast, our study used a sample of Spanish speakers from the global south. This contributes to addressing the challenge of sample diversity, ensuring that research encompasses a wide range of populations for a more comprehensive understanding (Kopal et al., 2023). The replication implemented in our study extends reported results to previously unexplored participant samples, thus contributing to bridging these gaps. However, we sought not only to replicate the task but also to extend and further characterize its findings. In this regard, we successfully replicated the task's main effect, which is the change in intensity rating when switching the attentional focus from arousing to non-arousing portions of unpleasant images. In this case, our effect size was *g* = 0.43 [0.06, 0.86], smaller than the one reported previously by Ferri et al. (2013), which was *g* = 1.4.

Several differences between our study and Ferri et al.’s could account for the difference in effect size. First, there are the global, general, and diffuse issues related to the repetition of the task that occur in a sample of individuals from a different country, year, language, and culture. We cannot know which of these factors is more important in this case, but they have been described as crucial in assessing cognitive and emotional processes (Barrett, 2012; Barrett et al., 2007; Gutchess & Rajaram, 2023; Immordino-Yang et al., 2016). A second set of differences is specific to the study and related to experimental design: we used a slightly larger sample (*n* = 55 compared to *n* = 41), which could account for some of the increase, as described (Loken & Gelman, 2017). Ferri et al. used a reduced rating scale (1 to 4 compared to ours, 1 to 9), which could also explain some of the increase, as it changed our effect from *g* = 0.4 to *g* = 0.5 when we remapped our ratings to the reduced scale. In addition, Ferri et al. asked participants for emotional intensity only, whereas we asked for both intensity and valence, which could have resulted in a larger cognitive load for our participants. And finally, in our case, participants were sitting in an experimental room, and in Ferri et al.’s study they were inside a magnetic resonance scanner, which can affect the behavioral outcomes of a task, most probably in a paradigm-specific manner (Assecondi et al., 2010; Koch et al., 2003; Kolodny et al., 2022; van Maanen et al., 2016).

Aside from effect size issues regarding our replication of the previous protocol, nevertheless, the fact that not only intensity but also valence shifted in the predicted direction (increase) when switching from arousing to non-arousing focus, maintaining an effect size of *g* = 0.4, reinforces the robustness of the result and provides evidence that the task provides an experimental instance of AD.

### AD Estimates

Our analysis of the AD task proposed an estimate of the AD capacity based on the difference in participants’ ratings between non-arousing and arousing focus conditions. We reasoned that the *change* in emotional rating, depending on the focus (i.e., attentional) condition, would constitute a behavioral readout of such an estimate. It is interesting to note that while our AD estimates clearly show that participants indeed regulate their emotions during the task and that they are consistent, the valence-based and intensity-based metrics show an important degree of inter-individual variability. We think that explaining this variability is an important task to better understand AD as a process. One way forward in this regard is to study the physiological correlates of behavioral readouts. For instance, several reports have shown a relationship between emotion regulation capacities and physiological markers such as heart rate variability (Min et al., 2023). Another interesting way to unpack this variability is considering personality traits or ER traits to explain how individuals respond to negative visual stimuli and down-regulate their emotions. Neuroticism, for example, has been described as the tendency presented by some individuals to experience negative emotions and distress (Ormel et al., 2013). Several studies have offered evidence suggesting that neuroticism is associated with specific patterns of gaze behavior toward negative emotional stimuli (e.g., Armstrong et al., 2010) and may influence selective attention (Richards et al., 2014). Unfortunately, no instruments are available to measure individuals' disposition to use AD as a strategy. This contrasts with other ER strategies, such as reappraisal and suppression, which have received more attention from the research community and have more established measurement tools (McRae & Gross, 2020). Future studies should develop tools that can contribute to measuring this ability.

### Behavioral Correlates of AD

We aimed to fill the gap in the current literature regarding a deeper characterization of AD. Concerning behavioral correlates, interesting data were found in terms of response time. Specifically, participants were faster to produce emotional ratings in the arousing focus condition compared to the non-arousing focus condition. This difference was evident for both intensity and valence ratings, with 60% and 67% of participants, respectively, showing faster responses for the arousing focus. The difference was larger for the valence rating, which is the first rating the participants had to report after watching IAPS images. Interestingly, we found associations between the ratings’ response times and the rating values, specifically for the arousing focus condition in the unpleasant images; higher intensity ratings were associated with shorter time responses, and conversely, lower valence ratings (more negative) were associated with faster response time. Furthermore, we observed that focusing attention on arousing portions of pictures, within unpleasant emotional stimuli, tends to prompt faster responses. This aligns well with previous studies, where faster recognition of emotional stimuli was associated with higher intensity and lower valence ratings, in the case of emotional faces (Sato & Yoshikawa, 2010), abstract emotional stimuli (Bartoszek & Cervone, 2022) and also of emotional events in experience sampling methodologies (Arndt et al., 2018). Overall, these data offer supporting evidence to the view that attentional focus is key to emotional experience and particularly to the process by which emotions are generated (Storbeck & Clore, 2007).

### Neuropsychological Correlates of AD

No robust associations were found when exploring the relationship between attentional capacities, evaluated through the ANT, and AD performance. Only performance on the Alerting Network exhibited a small and negative association with valence-based AD (*r* = -.33, *p* = .05) and a positive association with intensity-based AD (*r* = .31, *p* = 0.06). The Alerting Network has been closely linked to arousal (Petersen & Posner, 2012) and is described as responsible for achieving and maintaining an alert state. According to the review by Compton (2003), this network is crucial for processing emotional stimuli, as emotional states can enhance alertness and thus influence how attentional resources are allocated. Our data does not support the involvement of other attentional networks related to the *selection* of information, *shifting* attention (Orienting Network), or *resolving conflicts* amongst competing responses (Executive Network). One plausible explanation is that AD, as measured by our task, does not recruit these networks, since participants are simply instructed to fix their gaze on arousing and non-arousing areas of emotionally negative pictures. To explore further the relationship between AD and attentional abilities, future studies could replicate this task with other well-known neuropsychological tools that measure attention, such as the Paced Auditory Serial Addition Test (PASAT, Gronwall, 1977) or the Continuous Performance Test (CPT, Conners & Sitarenios, 2011). Another relevant area to explore is the assessment of AD in individuals with attentional disorders due to neurological damage, where AD impairment has been described (Salas et al., 2019).

### Conclusion

In conclusion, our study contributes to the growing body of literature on AD specifically, and emotion-cognition interaction more generally, by providing a detailed behavioral characterization of a task that measures this emotion regulation strategy. It also offers novel data regarding the attentional correlates of AD, suggesting a potential role for the Alerting Network. While some of our findings challenge existing assumptions about arousing focus conditions in AD paradigms, they also open new avenues for methodological refinement in this field. Future research should continue to investigate the cognitive and neural bases of AD, employing diverse measures and paradigms to unravel the complex interplay between attention, emotional reactivity, and emotion regulation.

## Supplementary Materials

**Table:** 

Type of supplementary materials	Availability/Access
Code	
Matlab code for controlling hardware and implementing task; and the code used to analyze data, produce plots, and compute statistics.	Salas et al. (2025a), Salas et al. (2025b)
Data	
Behavioral and eye-tracking data from attentional deployment task.	Rojas-Libano (2025)
Material	
Image files used as stimuli for task trials.	Salas et al. (2025a)

## Data Availability

All the data collected and used to compute the results presented in this article are available at Rojas-Libano (2025).
